# Water Dissociation on NiOOH in Alkaline Water Electrolysis Improves with Increasing Alkali Metal Cation Size

**DOI:** 10.1002/cssc.202402596

**Published:** 2025-04-26

**Authors:** Julia Gallenberger, Clara Gohlke, Marie Neumann, Anna K. Mechler, Jan P. Hofmann

**Affiliations:** ^1^ Surface Science Laboratory Department of Materials‐ and Geosciences Technical University of Darmstadt Peter‐Grünberg‐Straße 4 64287 Darmstadt Germany; ^2^ Electrochemical Reaction Engineering (AVT.ERT) RWTH Aachen University Forckenbeckstraße 51 52074 Aachen Germany

**Keywords:** alkali metal cations, oxygen evolution reaction, vibrational spectroscopy, water dissociation, X‐ray photoelectron spectroscopy

## Abstract

The activity of nickel‐based electrocatalysts toward the oxygen evolution reaction (OER) is influenced by the presence of alkali metal cations in the electrolyte. Since the underlying mechanism is not fully resolved yet, a study combining Raman, Fourier‐transform infrared, and photoelectron spectroscopies is conducted. It is found that an improved OER activity correlates with structural changes of the catalyst. The cations are adsorbed in increasing amounts in the order Li^+^ < Na^+^ < K^+^ < Cs^+^, opening the layers of the NiOOH layered double‐hydroxide structure and promoting a transition from a more β‐like to a more γ‐like NiOOH phase. In addition, the NiOOH surface gets increasingly deprotonated with increasing alkali cation size. The activated catalyst materials are stabilized in ultra‐high vacuum and exposed to controlled doses of H_2_O to analyze the catalyst–electrolyte interface in a quasi in situ approach with photoelectron spectroscopy. Going from Li^+^ to Cs^+^, more OH groups are found on the surface after the exposure to H_2_O, demonstrating that such structural changes are facilitating the dissociation of H_2_O. As the dissociation of H_2_O is a crucial step in many OER mechanisms, its modified efficiency can be correlated with the observed trends in OER activity in LiOH, NaOH, KOH, and CsOH.

## Graphical abstract

The activity of a nickel oxide‐based catalyst toward the oxygen evolution reaction is affected by the cations in the electrolyte. A higher current density in CsOH goes along with a more γ‐like NiOOH phase, a more deprotonated surface, and a facilitated water dissociation.

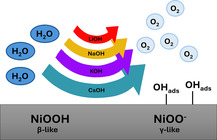

## 1 Introduction

For the electrolysis of water, the oxygen evolution reaction (OER) is the limiting half‐reaction, where the overpotential could not yet reach lower limits like they were achieved for the hydrogen evolution reaction.^[^ ^1^, ^2^ ^]^ To meet the performance demands of scalable and economical electrolyzer stacks for industry, there are many contributions in literature, which aim to improve the OER activity. A promising and economic materials class is transition metal oxides used in anodes for alkaline water electrolysis. These oxides are studied in various morphologies, elemental compositions, and electrochemical conditions.^[^ ^3^, ^4^, ^5^ ^]^ One parameter out of many that can influence the activity of the catalysts is the composition of the electrolyte. Ions in the electrolyte have a direct impact on the electric double layer (EDL).^[^ ^6^, ^7^, ^8^ ^]^ Its understanding has a pivotal role in understanding and tailoring materials in electrocatalytic systems. At the EDL, the surface of the OER active phase is in contact with the electrolyte. The precise structure of this EDL is influenced by the detailed surface structure of the catalyst, the kind of ions including solvation shells in the electrolyte as well as adsorbed at the electrode, and the interfacial pH, which can deviate from the bulk pH.^[^ ^9^ ^]^ In this study, we focus on the contribution of different alkali metal cations, whose effect on the EDL has been probed in various experimental and theoretical works.^[^ ^10^, ^11^, ^12^, ^13^, ^14^ ^]^ In the order Li^+^–Na^+^–K^+^–Cs^+^, the cation size increases, the electronegativity decreases, the hydration energy decreases,^[^ ^15^ ^]^ and the Lewis acidity decreases.^[^ ^16^ ^]^ These properties have implications on the hydration shell of these cations in the alkaline electrolyte. Taking Cs^+^ and Li^+^ for comparison, the solvation shell of Li^+^ is built from stronger non‐covalent bonds. Cs^+^, as a softer cation, in contrast, is less solvated and is, therefore, expected to adsorb in larger quantities on the electrode surface.^[^ ^9^, ^17^, ^18^ ^]^ Their hydration enthalpies range from 519 kJ mol^−1^ for Li^+^ to 409, 322, and 264 kJ mol^−1^ for Na^+^, K^+^, and Cs^+^, respectively.^[^ ^19^ ^]^ The surface of the catalyst, that is in our case the surface of a NiOOH, OER active phase, varies depending on the conditions. The NiOOH phase is unstable at open circuit potential, but is created when polarizing the Ni(OH)_2_ phase, of which two polymorphs exist, where α‐Ni(OH)_2_ is a hydrated, disordered form of β‐Ni(OH)_2_.^[^ ^20^, ^21^ ^]^ Charging of β‐Ni(OH)_2_ first leads to an oxidation of Ni^2+^ to Ni^3+^ and a phase change to β‐NiOOH. Upon further charging, the oxidation state of Ni increases up to Ni^3.66+^ and a γ‐NiOOH phase is formed. When starting with α‐Ni(OH)_2_, immediately γ‐NiOOH crystallizes upon polarization. In contrast to β‐NiOOH, in γ‐NiOOH both H_2_O and ions, including alkali metal cations, are intercalated.^[^ ^22^, ^23^, ^24^ ^]^ With X‐ray absorption spectroscopy (XAS), the intercalation of the cations was shown to be reversible when reducing the sample back to Ni(OH)_2_.^[^ ^24^ ^]^ Yet, the nuances of the phase transitions and structures are still a matter of debate.^[^ ^25^, ^26^ ^]^ For instance, Garcia et al.^[^ ^27^ ^]^ have shown the formation of NiOO^−^ at 1.7 V versus reversible hydrogen electrode (RHE) with Raman spectroscopy. The peroxo band increases when using different electrolytes from LiOH to NaOH, KOH, and finally CsOH. In the same manner, the current density increased during the OER. They propose that the NiOO^−^−M^+^ species acts as an intermediate of the OER. Such findings show that the surface structure of the NiOOH catalyst is complex, which complicates an evaluation whether the β‐NiOOH or γ‐NiOOH phase is more active toward the OER. Several studies exist claiming the β‐NiOOH phase to be the optimum phase, yet several other studies challenge that view and report higher activities of the γ‐NiOOH phase.^[^ ^28^, ^29^, ^30^ ^]^ In many more publications, the active phase has not been explicitly specified due to its instability under ex situ conditions, its often low crystallinity, disorder, and resulting difficulties obtaining high‐quality high‐resolution transmission electron microscopy and X‐ray diffraction data.^[^ ^20^, ^31^, ^32^, ^33^, ^34^ ^]^

Here, we used a combination of Raman spectroscopy, performed both in situ and ex situ, and X‐ray photoelectron spectroscopy (XPS) to track changes in the NiOOH phase induced by Li^+^, Na^+^, K^+^, or Cs^+^ in a purified (Fe‐free) electrolyte. Furthermore, the ultra‐high vacuum‐stabilized NiOOH phase was exposed to controlled doses of H_2_O, which is a quasi in situ approach to follow the electrolyte‐surface interactions. The outcome is followed with X‐ray and ultraviolet photoelectron spectroscopy (XPS, UPS). Our results imply that the improved OER activity in the order Cs^+^ > K^+^ ≥ Na^+^ > Li^+^ goes along with a more γ‐NiOOH like structure of the activated catalyst. These structural changes seem to be induced by an increased intercalation of the alkali cations in the same order, that is *c*(Cs^+^) > *c*(K^+^) > *c*(Na^+^) > *c*(Li^+^) after activation. Importantly, these alterations facilitate the dissociation of water. Both in the O 1*s* core level spectra and in the valence band spectra, a relative increase in adsorbed OH groups is observed, which is correlating with the activity of the catalyst. In many of the proposed mechanistic pathways for the alkaline OER, the dissociation of H_2_O is necessary for the formation of OER intermediates at the surface.^[^ ^35^, ^36^ ^]^ Therefore, the enhanced water dissociation has a direct effect on the overpotential required for the OER reaction.

## 2 Results and Discussion

**Figure 1:**
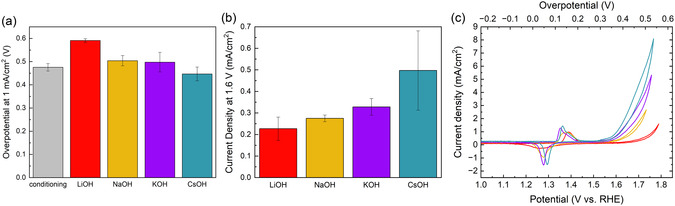
a) Overpotential at 1 mA cm^−2^, b) current density at 1.6 V versus RHE after activation in 1 m LiOH, NaOH, KOH, and CsOH. Before the activation, all samples were prepared with a conditioning protocol in 1 m KOH to form a Ni(OH)_2_ layer, that could then be activated in the respective alkali cation solutions. c) Cyclic voltammogram at 10 mV s^−1^ in 1 m LiOH (red), NaOH (yellow), KOH (purple), and CsOH (turquoise) from the extended activity measurements of the samples used for vibrational spectroscopy.

Ni plates were conditioned in purified 1 M KOH to obtain a Ni(OH)_2_ thin film (Figure S5, Supporting Information). The effectiveness of such an electrochemical conditioning to form Ni(OH)_2_ from Ni metal was demonstrated in a recent publication.^[^ ^37^ ^]^ Two sets of samples were prepared. One was used for XPS and UPS measurements, the other one for Fourier‐transform infrared (FTIR), Raman, and an extended activity measurement. The experimental sequence is outlined in Figure S1, Supporting Information. **Figure** **1**a shows the OER overpotential at 1 mA cm^−2^, referenced to 1.23 V. The gray bar shows the overpotential of the created Ni(OH)_2_ thin films measured in KOH at the end of the conditioning sequence. The samples were subsequently activated at 1.6 V versus RHE in 1 m LiOH, NaOH, KOH, and CsOH to form the OER active NiOOH phase. The electrochemical performance was evaluated with a chronopotentiometry (CP) measurement at 1 mA cm^−2^ (Figure 1a), chronoamperometry at 1.6 V vs. RHE (Figure 1b), and a CV up to 1.8 V versus RHE (Figure 1c) in the presence of the respective alkali hydroxide solutions. The activity follows the order LiOH < NaOH ≤ KOH < CsOH. This is in line with trends reported in literature.^[^ ^11^, ^27^, ^38^, ^39^ ^]^ In Figure S3, Supporting Information, the electrochemical activity is itemized for the two sets of samples used for XPS and vibrational spectroscopy, respectively.

**Figure 2:**
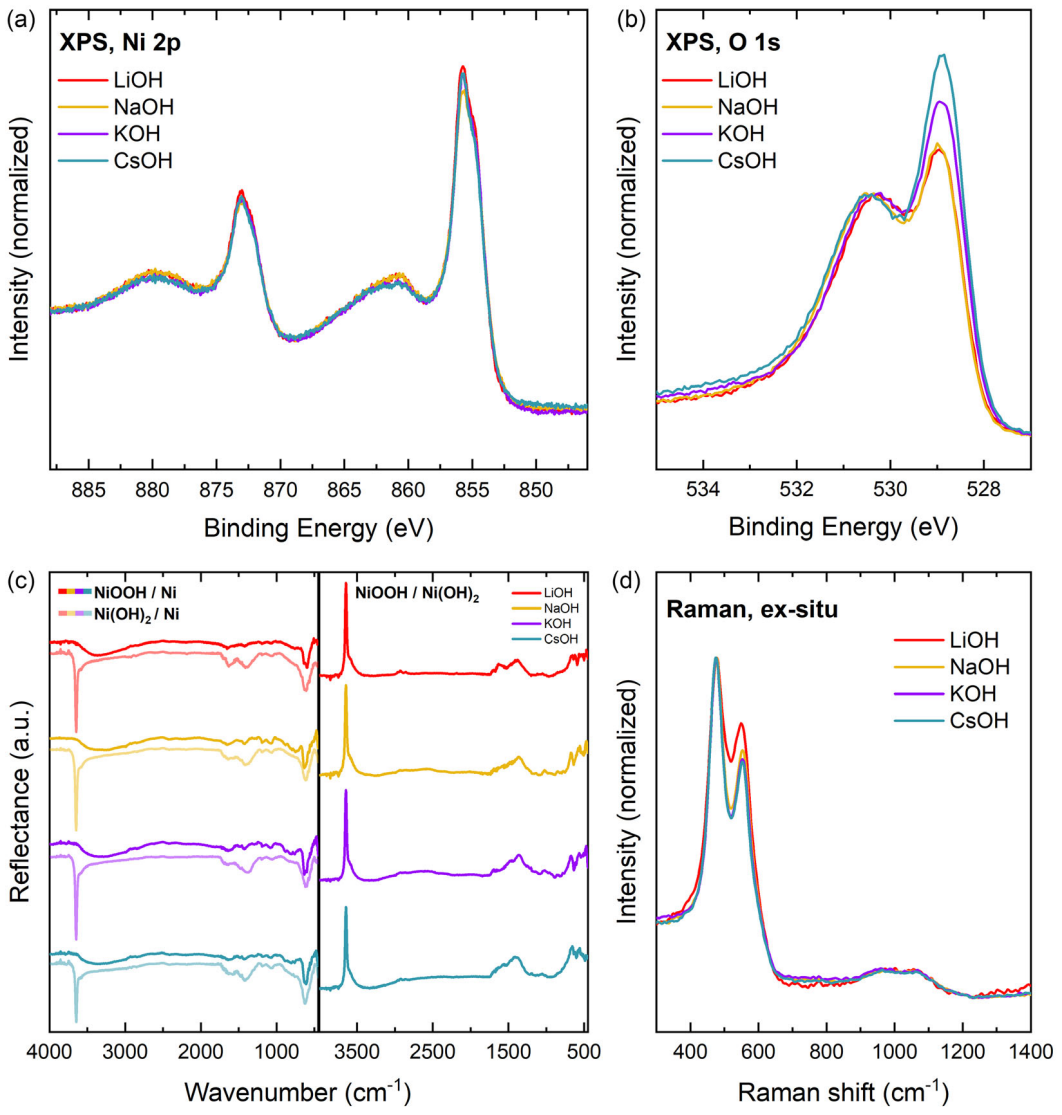
a) Ni 2*p*, b) O 1*s* core level spectra of NiOOH activated at 1.6 V versus RHE in LiOH, NaOH, KOH, and CsOH. c) Left: FT‐IRRAS of Ni(OH)_2_ prepared by conditioning (bright) and of NiOOH activated in the respective electrolyte (dark), right: difference spectra NiOOH/Ni(OH)_2_. d) Ex situ Raman spectra after activation.

The first set of activated samples was taken out of the electrochemical cell at 1.6 V versus RHE and transferred within 3 min into ultra‐high vacuum (UHV) to stabilize the phase. By then, some NiOOH can have decomposed into Ni(OH)_2_, but the majority of the sample is still in its NiOOH phase as formed during the electrochemical activation. This approach and the stability of the NiOOH phase were studied in a previous article.^[^ ^20^ ^]^ Additionally, a control experiment was performed, where the transfer time between activation with different alkali cations and vacuum stabilization was closely monitored. If the time period, during which the NiOOH decomposition is happening, is kept constant for all samples, the amount of decomposition is similar for all samples. Consequently, any differences between these spectra need to be an effect of the treatment with different cations. An extended discussion is done based on Figure S6, Supporting Information. The NiOOH phase shows the characteristic shape of the Ni 2*p* core level (**Figure** **2**a).^[^ ^20^, ^40^ ^]^ The valence band and core levels are shifted to lower binding energies compared to Ni(OH)_2_, indicating a p‐type electronic structure of the NiOOH phase. In our previous article, we could assign the peak at 528.9 eV in the O 1*s* spectrum to the unprotonated oxygen in the NiOOH structure. In the results presented in Figure 2b, the intensity of this peak depends on the type of electrolyte used during activation. Namely, it is largest after activation in CsOH, followed by KOH, and then NaOH and LiOH. The O 1*s* spectra are normalized at 530.4 eV. A similar trend was observed in the control experiment in Figure S6, Supporting Information, which confirms the cation effect on the amount of unprotonated oxygen on the surface. To understand this spectral evolution, a second set of samples was activated and transferred as quickly as possible into the FTIR sample compartment, which was evacuated for the FTIR reflection‐absorption spectroscopy measurement. Immediately afterward, a Raman spectrum was collected ex situ. Figure 2c compares the FTIR spectra of the Ni(OH)_2_ phase before activation, the NiOOH phase after activation, and the difference spectra with Ni(OH)_2_ as the reference spectrum. The spectra show that the phase composition before activation was similar for all samples, namely β‐Ni(OH)_2_, with a strong lattice OH stretching vibration at 3646 cm^−1^ and a smaller bending vibration at around 520 cm^−1^, and some α‐Ni(OH)_2_ with a lattice mode at 607–634 cm^−1^ and an OH bending vibration at 1380–1410 cm^−1^. Intercalated H_2_O contributes intensity at 3590 and 1630 cm^−1^. To conclude, applying the conditioning procedure to polished Ni metal creates mostly β‐Ni(OH)_2_ with some disorder appearing in the form of turbostratic α‐Ni(OH)_2_. This combination could signify some α/β‐interstratification.^[^ ^41^, ^42^, ^43^, ^44^ ^]^ After activation, the sharp peak at 3646 cm^−1^ is lost, which means the loss of non‐hydrogen‐bonded OH groups. This is partly caused by a general decrease of OH groups in NiOOH compared to Ni(OH)_2_, while the remaining OH groups are now bonded to hydrated H_2_O. The interfacial water appears in the spectra as a broad feature from 2670–3630 cm^−1^. An increased hydration of the material after activation in LiOH to CsOH is hardly visible in the difference spectra NiOOH/Ni(OH)_2_. However, this could be caused by difficulties in correctly determining the gradient of the baseline in these spectra. In the FTIR spectra, no trend can be derived that correlates with the variation of the electrolyte. The situation is different for Raman spectroscopy. Here, Figure 2d displays the *E*_g_ and *A*_g_ lattice vibrations of NiOOH at about 477 and 555 cm^−1^, respectively, and some intensity from 900 to 1160 cm^−1^, which is assigned to NiOO^−^.^[^ ^45^, ^46^ ^]^ The ratio of the *E*_g_ and *A*_g_ lattice vibrations was found to be sensitive to the structure of NiOOH, whereby a higher peak ratio was found for γ‐NiOOH.^[^ ^46^, ^47^ ^]^ In Figure 2d, a correlation of the peak ratio with the kind of electrolyte becomes apparent. The spectra were normalized to visualize the relative increase of the 555 cm^−1^ band after activation in LiOH compared to after activation in CsOH, signifying an increasing amount of γ‐NiOOH with increasing cation size. The peak of the *E*_g_ lattice vibration shifts from 477.4 to 475 cm^−1^ after activation in LiOH versus in CsOH.

**Figure 3:**
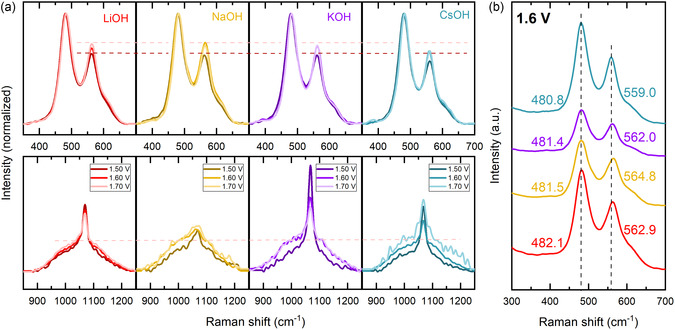
a) Normalized in situ Raman spectra at increasing potentials in different electrolytes. b) Stacked spectra recorded at 1.6 V versus RHE. Color code: LiOH: red, NaOH: yellow, KOH: purple, CsOH: turquoise.

Since the NiOOH phase is slowly decomposing to Ni(OH)_2_ during the measurement in air, Raman spectra were also acquired in situ. **Figure** **3**a shows that with increasing potential, the peaks shift to higher wavenumbers and the peak ratio changes, irrespective of the electrolyte. The peak ratio change with elevating polarization was already observed in previous publications.^[^ ^46^, ^47^ ^]^

In the upper row of Figure 3a, the peak ratio is compared for in situ measurements in the various electrolytes. Dashed lines with the peak maxima in LiOH as reference are included to visualize the lower intensity at 555 cm^−1^ in CsOH compared to the other electrolytes, which fits to the trend seen in the ex situ Raman measurements (Figure 2d). The lower row in Figure 3a shows the spectral region of NiOO^−^. The sharp peak at 1068 cm^−1^ is attributed to carbonate in the electrolyte.^[^ ^48^ ^]^ Garcia et al.^[^ ^27^ ^]^ observed a cation dependence of this region. As its intensity increases the most in CsOH electrolyte in their study, they correlate this to the increased OER activity in CsOH. In our spectra, the intensity in this region increases the most during potential increase in CsOH. Comparing the total area of the spectra obtained in different electrolytes at 1.7 V versus RHE, there is a slight increase from LiOH to NaOH, KOH, and finally CsOH.

However, this trend is not reproduced for the measurements at lower potentials or ex situ. Possibly, the stabilization of NiOO^−^ by the cations is stronger on the surface than in the bulk. In this case, surface‐enhanced Raman spectroscopy, which was not employed here, might yield more precise results. Yet another observation in the Raman spectra was that the peaks shifted to lower wavenumbers from LiOH to CsOH (Figure 3b). This was also observed by Garcia et al.^[^ ^27^ ^]^ Michael et al.^[^ ^38^ ^]^ and Hou et al.^[^ ^13^ ^]^ and is related to a longer Ni—O bond with decreasing wavenumbers. To sum up the findings, NiOOH undergoes structural changes when activated with different alkali metal cations. With this knowledge, we want to come back to the O 1*s* spectrum and discuss the assignment of the peak at 528.9 eV. Payne et al.^[^ ^49^ ^]^ show in their O 1*s* spectra a smaller O^2−^ peak in β‐NiOOH than in γ‐NiOOH. This enhanced deprotonation of NiOOH in the γ‐phase is consistent with the higher oxidation state of Ni found in this phase. Combined with our results from Raman spectroscopy, we can confidently argue that the change in peak ratio in our O 1*s* spectra in Figure 2b is due to a structural change of a more β‐like NiOOH to a more γ‐like NiOOH, when changing the electrolyte from LiOH, to NaOH, KOH, and finally CsOH.

**Figure 4:**
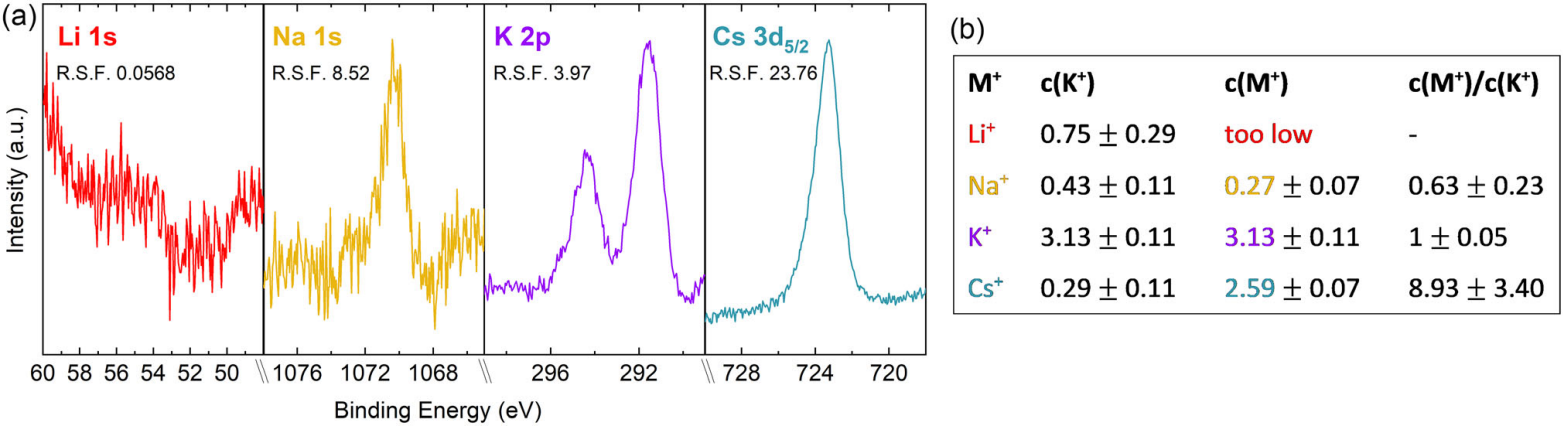
a) Core level spectra of alkali metal cations adsorbed on NiOOH activated at 1.6 V versus RHE. b) Calculated at% of the respective alkali metal cations. Orbitals used for calculation: Ni 2*p*, O 1*s*, C 1*s*, K 2*p*, Na 1*s*/Cs 3*d*.

We now address the question what might cause this structural transformation. XPS allows for a quantitative analysis of the elemental surface composition of the samples, which we used to determine the amount of alkali metal cations adsorbed on the surface or intercalated in the outer layers of the films. For the stoichiometry calculations, we used the core level spectra Li 1*s*, Na 1*s*, K 2*p*, C*s* 3*d* for the alkali metal cations (**Figure** **4**a), Ni 2*p* and O 1*s* for the NiOOH catalyst, and C 1*s* to include carbon residuals, employing the relative sensitivity factors of Scofield.^[^ ^50^ ^]^ Figure 4b lists the calculated values. During the activation, the respective alkali metal cations are adsorbed, where *c*(Cs^+^) > *c*(Na^+^) > *c*(Li^+^). The exact concentration of Li^+^ was not calculated as the core level spectrum hardly shows any intensity (Li also has a very low photoionization cross section). In the second column of Figure 4b the concentration of K^+^ was included because it shows that some K^+^ is left in the structure after the conditioning in 1 m KOH. It also shows that Cs^+^ is most effective in replacing K^+^, since *c*(K^+^)_CsOH_ < *c*(K^+^)_NaOH_ < *c*(K^+^)_LiOH_ after activation. Actually, when analyzing Ni(OH)_2_ after conditioning, very little K^+^ appears in XPS, with values of 0.1–0.2 at%. The increase of K^+^ in the XPS spectrum after activation in LiOH, NaOH, and CsOH, needs to either stem from K^+^ traces in the respective electrolyte or from K^+^ that was built into the bulk of the Ni(OH)_2_ structure during conditioning and was enriched at the surface during the activation. This is an indication that the intercalation or adsorption of alkali metal cations is quasi reversible when the NiOOH phase is reduced to Ni(OH)_2_ inside the electrolyte, as it was also observed with in situ XAS by Trzesniowski et al.^[^ ^24^ ^]^

In a next step, we exposed the activated catalyst to controlled doses of H_2_O to study by XPS and UPS if the adsorption or dissociation of water is influenced by the type of cation in the electrolyte. This is a quasi in situ approach to test any impact on the energetics of the OER mechanism. There are several possible pathways for the oxygen evolution reaction.^[^ ^51^, ^52^ ^]^ Many of those include one step, where H_2_O needs to dissociate to form an OER intermediate at the catalyst surface.^[^ ^53^, ^54^ ^]^

**Figure 5:**
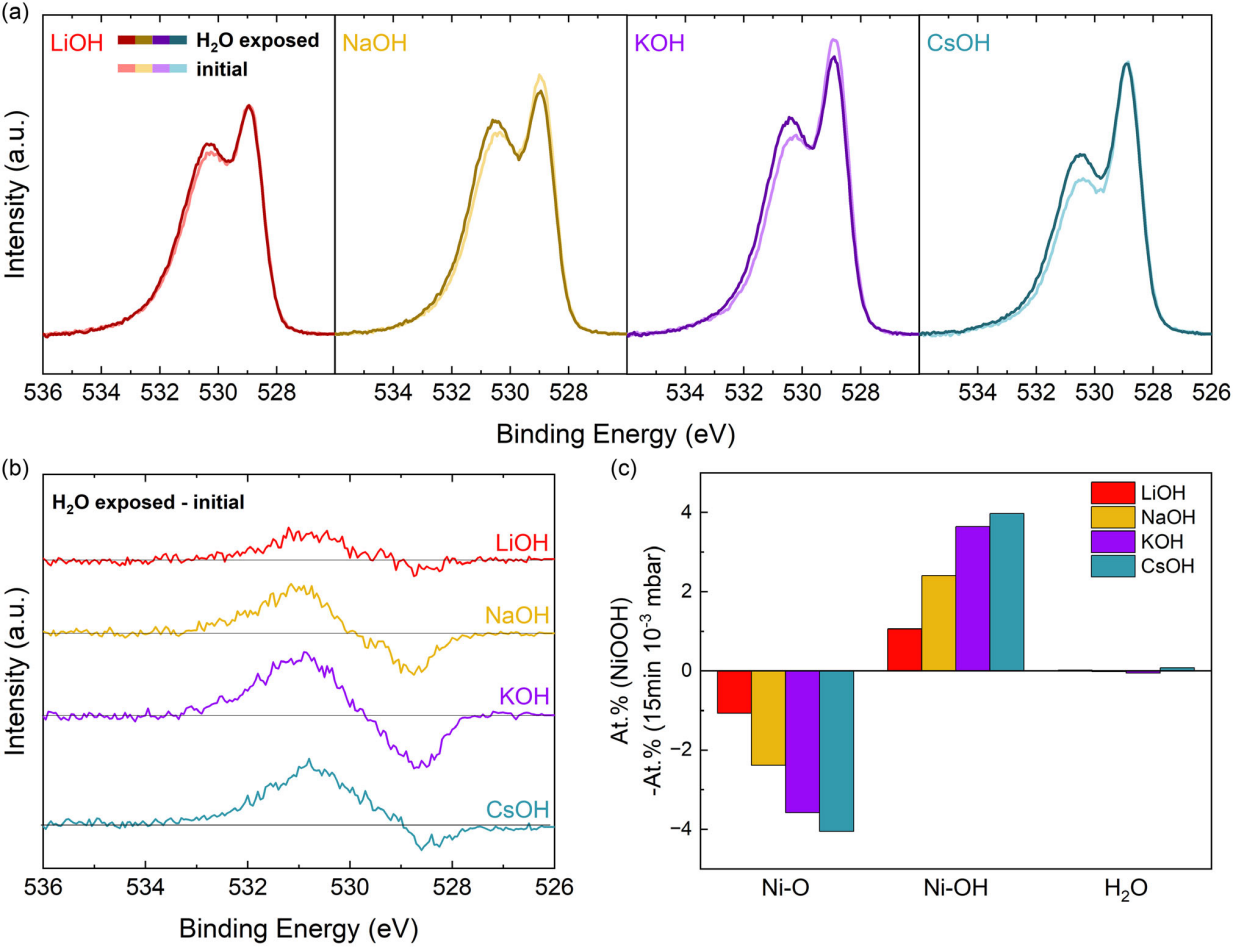
a) O 1*s* spectra of NiOOH, which was activated in different alkali metal cation electrolytes, stabilized in UHV (“initial”), and exposed inside UHV to H_2_O (“H_2_O exposed”). b) Difference spectra of “H_2_O exposed”‐“initial” shown in (a). c) Change in at% of O bonded to Ni, OH bonded to Ni, and H_2_O, obtained by a fitting of the spectra in (a).

In the experiment, the vacuum‐stabilized NiOOH phase was exposed inside UHV to a dose of 15 s·10^−3^ mbar H_2_O, measured with XPS and UPS, exposed again to H_2_O, but now for 15 min·10^−3^ mbar, and measured again with XPS and UPS. The changes that are observed are gradual. Therefore, only the initial and the final spectra, after 15 min·10^−3^ mbar, will be shown in the following plot. For easier readability, the final spectra will be denoted as “H_2_O exposed.” The H_2_O exposure was done at room temperature. Water in contact with the surface will either adsorb as H_2_O molecule, dissociate, or not react with the surface at all. **Figure** **5**a shows the O 1*s* spectra directly after stabilization in vacuum and after exposure to H_2_O for each electrolyte. What they have in common is an increase of the peak at 530.5 eV and a slight shift of this peak to higher binding energies. Figure 5b displays the difference spectra “H_2_O exposed”‐“initial”. For Figure 5c, the O 1*s* was fitted with one peak at 528.8 eV for O bonded to Ni, one peak around 530.4 eV for OH bonded to Ni, and one small peak around 532.8 eV for molecular H_2_O. An exemplary fit is shown in Figure S7, Supporting Information. The relative change of these components with H_2_O exposure is pictured in Figure 5c. In all of those illustrations it is visible, that the increase of OH groups after H_2_O exposure scales with the electrolyte cations in the order Li^+^ < Na^+^ < K^+^ < Cs^+^. Simultaneously, the at% of Ni—O decreases, while there are no significant changes in the component assigned to adsorbed or intercalated H_2_O.

**Figure 6:**
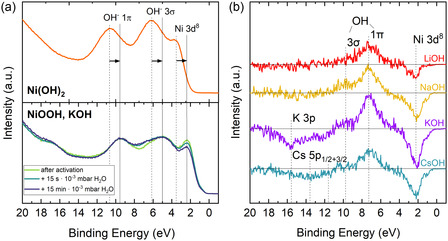
a) Top: He II valence band (VB) spectrum of Ni(OH)_2_. Bottom: He II VB spectrum of NiOOH activated in 1 m KOH in the stabilized state and exposed to H_2_O. b) Difference spectra of the H_2_O exposed surface minus the initial surface for all electrolytes.

To probe adsorbates on the outmost surface, UPS is a suitable method with a detection depth of 20 Å at most.^[^ ^55^ ^]^ **Figure** **6**a shows the valence band, measured with monochromatic He II radiation (40.8 eV), of Ni(OH)_2_ and NiOOH formed in 1 m KOH. The three peaks in the valence band of Ni(OH)_2_ stem from a Ni 3*d* ^8^ emission, and from the 3*σ* and 1*π* orbitals of the OH groups in Ni(OH)_2_.^[^ ^56^ ^]^ In NiOOH these peaks appear as well, just shifted by 1.1 eV to lower binding energies due to a Fermi level shift. Note, that these measurements were not done in situ and that the present valence band spectrum should not be taken as a reference for a clean and well‐structured NiOOH surface. In this study, the important finding is that upon exposure to H_2_O, there is an intensity increase at 6–8 eV and a decrease at 2.4 eV. To interpret these changes, difference spectra were calculated and are plotted in Figure 6b. In this representation, it becomes visible that the intensity increase from 6–8 eV is actually composed of 2 peaks. Those can be assigned to the 3*σ* and 1*π* orbitals of OH groups adsorbed onto the surface. These add to the maximum detection depth of UPS, which is the cause for the decrease of the Ni 3*d* ^8^ emission at 2.4 eV and the decrease of the signal of the K 3*p* and Cs 5*p* core levels in the case of activation in KOH and CsOH, respectively.^[^ ^57^ ^]^ The adsorption of OH recognized in the valence band fits to the increase of OH groups in the O 1*s* core level spectra. Similarly, a cation dependence is also visible in the valence band spectra, where NiOOH activated in LiOH adsorbs the least OH groups on the surface.

We have shown that structural changes and a deprotonation of the NiOOH surface upon interaction with different alkali metal cations cause an improved dissociation of H_2_O. A favored dissociation also implies a modified adsorption‐free energy and an enhanced stability of the dissociation products on the catalyst surface. Their adsorption is part of most mechanisms to form key OER intermediates. For instance, the reaction
(1)

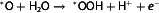

is the rate and potential determining step (RDS/PDS) in many calculations.^[^ ^35^, ^36^ ^]^ In alkaline solution, this step is written as^[^ ^58^ ^]^

(2)

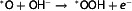




The bond strength between metal center to adsorbed *OH is often discussed in the context of volcano plots. According to the Sabatier principle, an intermediate should neither bond too weakly nor too strongly to the surface. It was found that in the OER mechanism, the adsorption energy of O* scales linearly with the adsorption energies of HO* and HOO*. Additionally, a universal scaling relationship between the free energy of adsorption of *OH and *OOH was found to be at Δ*G*_OH_ + Δ*G*_OOH_ = 3.2.^[^ ^59^ ^]^ In volcano plots, catalysts with well–balanced adsorption energies of all intermediates, show the best catalytic performance.^[^ ^60^ ^]^ Enhanced adsorption of OH, as it is observed in Figure 5 and 6, can therefore alter the energy of the rate and potential determining step of the OER and thereby lower the overpotential both in acidic and alkaline solution.

Hou et al. have already calculated a higher OH adsorption energy in the presence of Cs^+^.^[^ ^11^ ^]^ In the present study, we could verify this result experimentally.

What exactly causes the changes in dissociation activities of H_2_O and the associated improvement of the OER? Our article and the works of others have shown that NiOOH undergoes structural transformations depending on the electrolyte cations. First, there are variations of Ni—O bond lengths. Raman results show they are elongated in CsOH, whereas XAS measurements indicate the opposite.^[^ ^11^, ^27^, ^38^, ^61^ ^]^ We speculate that Cs^+^ is attracting charge of the lattice oxygen, whereby the Ni—O bond length is increased in Raman spectroscopy. A new finding is that the peak ratios of the *E*_g_ and *A*_g_ lattice vibrations of NiOOH in the Raman spectra are also affected. The peak ratio rises from LiOH to CsOH, which signifies a structural change to a more γ‐like NiOOH. This phase has a more open structure compared to β‐NiOOH, as it is intercalated with H_2_O and ions. This result supports the calculations of Görlin et al. who calculated an increase in layer separation from Li^+^ to Cs^+^. The cause is an increased cation‐oxygen coordination distance of the cations both with lattice oxygen and nearby water molecules.^[^ ^61^, ^62^ ^]^ We could interpret that the intercalation is directly visible in the XPS core level spectra of the alkali metal cations, where more at% of Cs^+^ than of Li^+^ have been detected in the surface near layers. Yet, here, we cannot specify how much of the signal is from intercalated or surface‐adsorbed cations. We expected to see the intercalation of H_2_O in the FTIR spectra, however, no unambiguous correlation of the spectra with the electrolyte could be found. The O 1*s* core level spectra show that the structural change is accompanied by more deprotonated sites after activation in CsOH. This makes sense, as Ni was found to have a higher oxidation state in γ‐NiOOH.^[^ ^63^ ^]^ Furthermore, from the perspective of charge compensation, the presence of, e.g., Cs^+^ on the surface can contribute to the deprotonation. As Cs^+^ has a weaker solvation shell, it is able to interact with and adsorb on the surface more effectively than the small, harder, hence stronger solvated, Li^+^.^[^ ^17^, ^18^ ^]^ The abundance of deprotonated sites could be a major factor in the enhanced dissociation of H_2_O. Zaffran and Toroker have calculated that the formation of the OOH intermediate, that forms with the dissociation of H_2_O, requires less energy on a deprotonated NiO_2_ surface than on a partly hydrogenated NiOOH_*x*_ surface. They argue that an electrostatic repulsion of the OOH intermediate with the H atoms on the catalyst surface destabilizes the OOH group.^[^ ^64^ ^]^ Furthermore, the deprotonation promoted by the alkali metal cation could first increase the Ni oxidation state and then favor a charge transfer from O to Ni. Both outcomes, an elevated oxidation state of Ni or an electron‐deficient oxygen site, were shown to enhance the OER.^[^ ^65^, ^66^, ^67^, ^68^, ^69^ ^]^ We also want to reflect on the findings of Görlin et al. who correlate the increase in OER activity from LiOH to CsOH with an increase of pH of these solutions.^[^ ^61^ ^]^ Since we see an increased adsorption of the larger cations and a correlating structural transformation of NiOOH, we believe that the cations do have their own contribution to the OER activity. However, the change in pH can very well have an additional and significant influence, especially when discussing the deprotonation of the catalyst surface. The pH of the solutions used in this study is displayed in Table S3, Supporting Information.

## 3 Conclusion

To summarize, we demonstrate that the activation of a nickel oxide OER catalyst in LiOH, NaOH, KOH, or CsOH affects both the structure and the surface of the OER‐active NiOOH phase. After activation, increasing amounts of alkali metal cations are adsorbed on the catalyst in the order Li^+^ < Na^+^ < K^+^ < Cs^+^, whereby the layers of the NiOOH layered double hydroxide structure are opened up to form a more γ‐like NiOOH phase. Additionally, from LiOH to CsOH an increased deprotonation of the NiOOH surface is observed. Importantly, a more deprotonated surface is shown to result in a more effective dissociation of water on that surface. As the dissociation of water is a crucial step in many OER mechanisms, an improved H_2_O dissociation might directly correlate with the observed decreased OER overpotential.

Yet, the discussion highlights that the facilitation of H_2_O dissociation can be a result of many intertwined phenomena. Structural bulk and surface changes, pH changes, changes in the electric double layer, and probably even more aspects need to be considered in future studies about the impact of alkali metal cations on nickel‐based anodes for the alkaline water electrolysis.

## 4 Experimental Section

4.1

4.1.1

##### Sample Preparation

Nickel plates of 12 mm diameter and 1 mm thickness (Goodfellow, 99.0% purity) were used as substrate. After polishing with 0.5 μm alumina paste (MicroPolish Alumina, Buehler), they were cleaned sequentially in an ultrasonic bath with acetone, isopropanol, and Millipore water for 5 min each. Next, a thin layer of Ni(OH)_2_ was formed by electrochemical conditioning, as described in the next section.

##### Electrochemical Measurements

Ex situ electrochemical measurements were conducted in a three‐electrode setup in a PECC‐2 cell from Zahner Elektrik, controlled by a potentiostat from Gamry Instruments (Interface 1000E). Before the measurement, a Hg/HgO reference electrode from ProSense was calibrated against a RHE (HydroFlex, Gaskatel). The Hg/HgO potential in 1 m KOH at RT varied between 0.913 and 0.921 V versus RHE. The counter electrode was made of a platinum wire. Via electrochemical impedance measurements at open circuit potential (OCP), the current responses during cyclic voltammetry (CV) were manually *iR*‐corrected in the data post‐treatment. All samples were conditioned in purified 1 m KOH (Carl Roth, ICP‐OES: <0.6 ppb Fe). The purification was done according to the article of Spanos et al. and its effectiveness was confirmed with inductively coupled plasma optical emission spectrometry (ICP‐OES) (Table S1, Supporting Information).^[^ ^70^ ^]^ The conditioning was done for 4 h, which means 650 CV from 0.5–1.6 V versus RHE with 100 mV s^−1^ scan rate. The full protocol is described in Figure S2a, Supporting Information.

The conditioned samples were then used to study the impact of the alkali metal cations. For this purpose, they were activated in the respective 1 m MOH solution (M = Li, Na, K, Cs). The activation was always performed 1 day after the conditioning. In between the samples were stored in air. Since all samples had a conditioning pretreatment in 1 m KOH, some K^+^ remained on the samples. The catalyst surface was therefore influenced by a combination of M^+^ and K^+^, that is the respective cation from the electrolyte plus potassium from the sample preparation.

The following electrolytes were used: 1 m LiOH (anhydrous, 99.9% trace metals basis, Sigma‐Aldrich), 1 m NaOH (1 N, volumetric standard solution, Carl Roth), 1 m KOH (1 N, volumetric standard solution, Carl Roth), 1 m CsOH (cesium hydroxide monohydrate, 99.95% trace metals basis, Sigma‐Aldrich). 1 m LiOH and 1 m CsOH were prepared with Millipore water and the molarity was verified by titration. All electrolytes were purified using the method by Spanos et al.^[^ ^70^ ^]^ yielding Fe contents ≤1 ppb measured with ICP–OES. The activation consisted of chronoamperometry (CA) at 1.6 V versus RHE for 2 × 10 min. Before and after activation, the OER activity was measured using CV and CP. The full protocol is again outlined in Figure S2b, Supporting Information. After the last CA step, the sample was removed from the electrolyte while the potential was still applied. The sample was rinsed with ultrapure water and transferred as quickly as possible to the ex situ spectroscopy. A first set of samples was stabilized within 3 min in UHV for XPS and UPS measurements. A second set was freshly prepared and activated and likewise transferred within 3 min to the FTIR sample compartment and evacuated to *p* < 3 mbar, then transferred to the Raman microscope for the ex situ and in situ characterization. After the vibrational spectroscopy characterization, an extended activity measurement of the samples was added. The detailed procedure is outlined in Figure S2c, Supporting Information. All experiments were performed at RT.

##### X‐Ray and Ultraviolet Photoelectron Spectroscopy

For the XPS measurements, a SPECS PHOIBOS 150 spectrometer implemented at the DAISY‐FUN cluster tool was used. It is equipped with an Al K_α_ X‐ray source (monochromatic Focus 500 with XR50 M (SPECS), hv = 1486.74 eV). Survey and detail spectra were measured in fixed analyzer transmission mode, while choosing a pass energy of 20 eV (step size of 0.5 eV) for the survey and 10 eV (step size of 0.05 eV) for the core levels. The system was calibrated to 0.00 eV binding energy of the Fermi level of sputter‐cleaned Au and Cu as well as to the emission lines of Au 4*f*_7/2_ at 83.98 eV, Ag 3*d*_5/2_ at 368.26 eV, and Cu 2*p*_3/2_ at 932.67 eV binding energy with deviations ≤0.1 eV. The valence band spectra were measured with UPS, using a helium lamp (HIS 13 Mono, Focus GmbH) with monochromatized HeII radiation of hv = 40.81 eV. Here, a pass energy of 5 eV and a step size of 0.05 eV was chosen. The data analysis was performed with CasaXPS, version 2.3.22.^[^ ^71^ ^]^ The O 1*s* core level spectra were fitted with a Shirley background and peaks of a GL(30) line shape. Intensity calculations were done based on relative sensitivity factors published by Scofield.^[^ ^50^ ^]^

##### Raman Spectroscopy

Spectra were acquired with a Bruker Senterra I Raman microscope with a 532 nm laser. Ex situ measurements were performed at 9–18 cm^−1^ resolution, 50x objective, a laser power of 37 mW on the sample surface, and 5 s integration time with 2 coadditions. For in situ measurements, a commercial cell named “TSC Raman” from rhd instruments was used. It was operated in a three‐electrode configuration. As counter electrode, a gold‐plated ring was installed in the base, and as reference electrode, a leak‐free micro Ag/AgCl electrode from Innovative Instruments was used and calibrated against a reversible hydrogen electrode (RHE, HydroFlex, Gaskatel). Potential control was performed with a potentiostat from Gamry instruments (Interface 1000E). The potential was increased in steps of 0.05 V from 1.20 to 1.50 V, followed by steps of 0.02 V from 1.50 to 1.70 V versus RHE. At each step, the potential was applied for 110 s while the Raman spectrum was taken after 60 s of applied potential. Here, the 20x objective was chosen, with a laser power of 36 mW reaching the sample surface, and again 5 s integration time with 2 coadditions was set. For plotting and interpretation of the in situ Raman spectra, a linear background was subtracted in the regions of interest.

##### FTIR Spectroscopy

FTIR spectroscopy was done on a VERTEX 80v FTIR spectrometer (Bruker) from 4000 to 400 cm^−1^. A room temperature DLaTGS detector was employed. Samples were measured on a monolayer/grazing angle specular reflectance accessory (Specac) at an incidence angle of 70° w.r.t. the surface normal. For background scans, a bare, polished nickel surface was used. Both sample and background scans (250 scans each) were acquired with a scanner velocity of 2.5 kHz and a resolution of 4 cm^−1^.

## Conflict of Interest

The authors declare no conflict of interest.

## Author Contributions

**Julia Gallenberger**: conceptualization, investigation, visualization, writing—original draft. **Clara Gohlke**: development of conditioning protocol, writing—review & editing. **Marie Neumann**: investigation ICP‐OES, writing—review & editing. **Anna K. Mechler**: supervision, funding acquisition, writing—review & editing. **Jan P. Hofmann**: writing—review & editing, supervision, conceptualization, funding acquisition.

## Supporting information

Supplementary Material

## Data Availability

The data that support the findings of this study are openly available in Zenodo at https://doi.org/10.5281/zenodo.15174433, reference number 15174433.
