# Supplementary material for: Water Dissociation on NiOOH in Alkaline Water Electrolysis Improves with Increasing Alkali Metal Cation Size
Source: ChemSusChem. 2025 Apr 26;18(13):e202402596. doi: 10.1002/cssc.202402596 (PMC12232099; doi:10.1002/cssc.202402596)
Supplement: Supplementary file 1 — Supplementary Material [file CSSC-18-e202402596-s001.pdf]

Supporting Information

Water dissociation on NiOOH in alkaline water electrolysis improves with increasing alkali metal cation size

Julia Gallenberger[a], Clara Gohlke[b], Marie Neumann[a], Anna Mechler[b], Jan P. Hofmann[a]

[a] J. Gallenberger, M. Neumann, Prof. J. P. Hofmann
Surface Science Laboratory, Department of Materials- and Geosciences, Technical University of Darmstadt, Peter-Grünberg-Straße 4, Darmstadt, Germany
E-mail: jgallenberger@surface.tu-darmstadt.de, hofmann@surface.tu-darmstadt.de

[b] C. Gohlke, Prof. A. Mechler
Electrochemical Reaction Engineering (AVT.ERT), RWTH Aachen University, Forckenbeckstraße 51, 52074 Aachen, Germany

Experimental sequence and electrochemical protocols

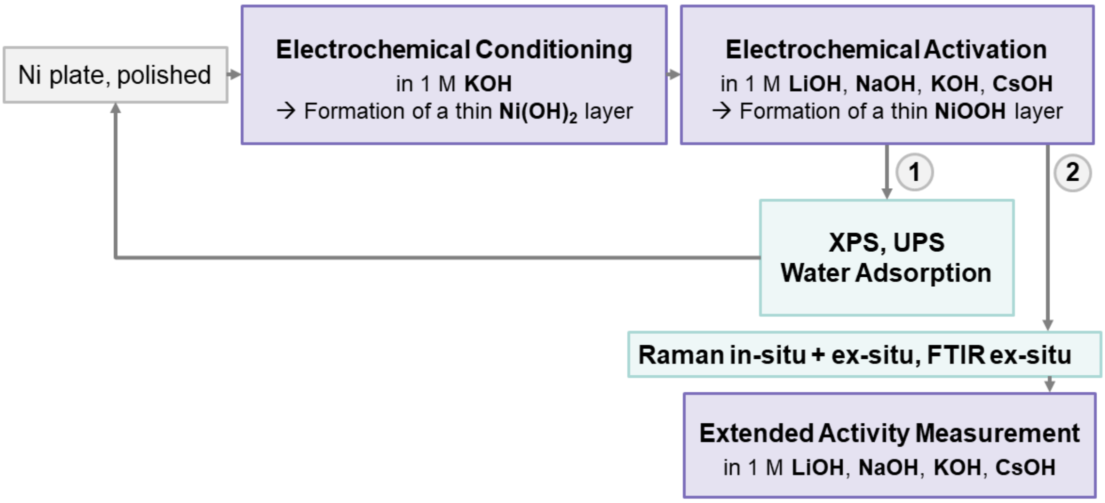

Figure S1. Experimental sequence of the study. The details about the electrochemical measurements are illustrated in Figure S2.

Figure S1 outlines the major experimental steps done in the study as described in the main paper. In Figure S2 the details of the electrochemical sequences are illustrated. In Figure S2b, the blocks electrolyte resistance, activity measurement, and activation were measured two times in a row. After the second activation the potentiostat was switched off at 1.6 V vs. RHE and the sample was transferred to the spectroscopy analysis.

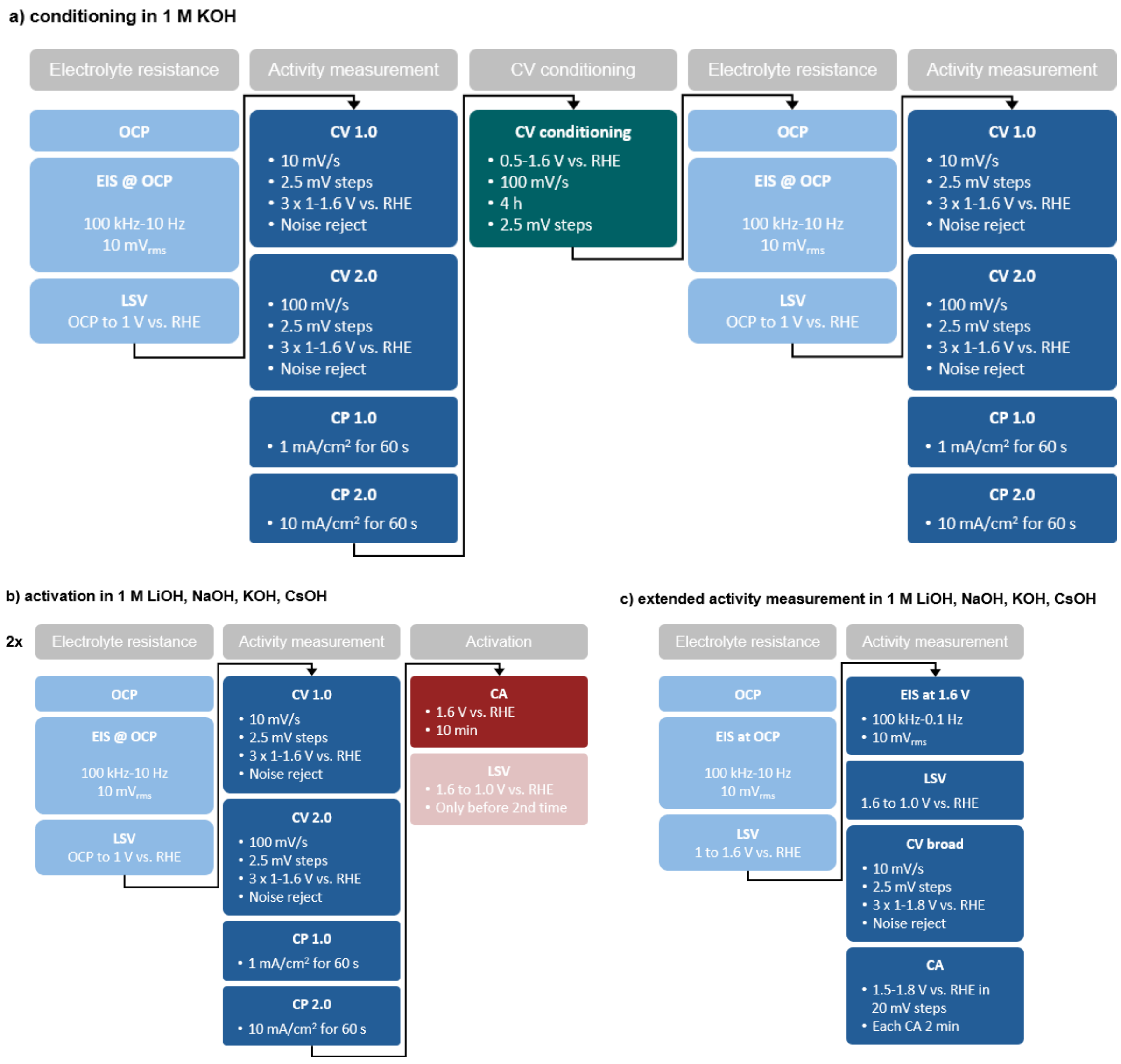

Figure S2. Electrochemical protocol of a) the conditioning, to form the $Ni(OH)_2$ phase, b) the activation, to form the NiOOH phase, c) an extended activity measurement.[37]

Electrochemical measurement results

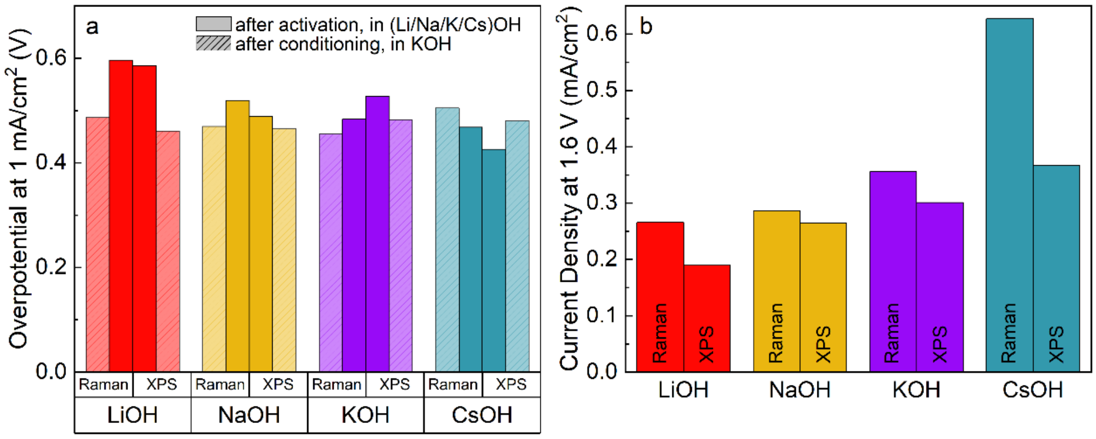

Figure S3. a) Overpotential at 1 mA/cm^2 after conditioning and activation, b) current density at 1.6 V vs. RHE after activation. The left and right bars are the results from samples taken for analysis with Raman and FTIR (labelled “Raman”) and XPS, respectively.

Purification of the electrolytes

Table S1. Fe concentration measured with ICP-OES before and after purification.

**Sample**	**Fe concentration (in ppb)**
Ultrapure Water	< 1
Millipore Water	1 +/- 0.2
LiOH	5.6 +/- 0.1
**LiOH purified**	**< 1**
NaOH	22.5 +/- 0.2
**NaOH purified**	**< 1**
KOH	5.8 +/- 0.2
**KOH purified**	**< 1**
CsOH	95.5 +/- 0.6
**CsOH purified**	**< 1**

The ICP-OES measurements of the electrolytes were performed using an iCAP PRO instrument from ThermoFischer. The measurement was performed in axial mode, and the uptake time of the electrolyte to the Ar humidifier was 60 seconds, with a rinse time of 30 seconds. The intensities of three different wavelengths (Fe 259.940 nm, Fe 239.562 nm, Fe 238.204 nm) were used for the ICP-OES measurements. An average value is shown in the table. At each wavelength, three separate measurements were performed and then averaged. The intensities were then compared with the calibration curves, and the following concentrations were calculated for the electrolytes, as seen in Table 1. The calibration was performed with Fe concentrations for the Standards from 2 ppb to 100 ppb. The standard solution was 3% ultrapure HNO_3 in 10 ml ultrapure water. It has to be taken into account, that due to the low Fe concentrations in the sample solutions an accurate measurement is difficult. Still the trend of a large reduction in Fe concentration is clearly visible.

Morphology of $Ni(OH)_2$

The morphology of the prepared $Ni(OH)_2$ thin films was measured with scanning electron microscopy (SEM). For the SEM experiments a MIRA3-XM microscope from TESCAN was used with an in-lens SE detector.

The $Ni(OH)_2$ layer that was formed from Ni metal via the conditioning protocol, shows a flake-like structure in the SEM images.

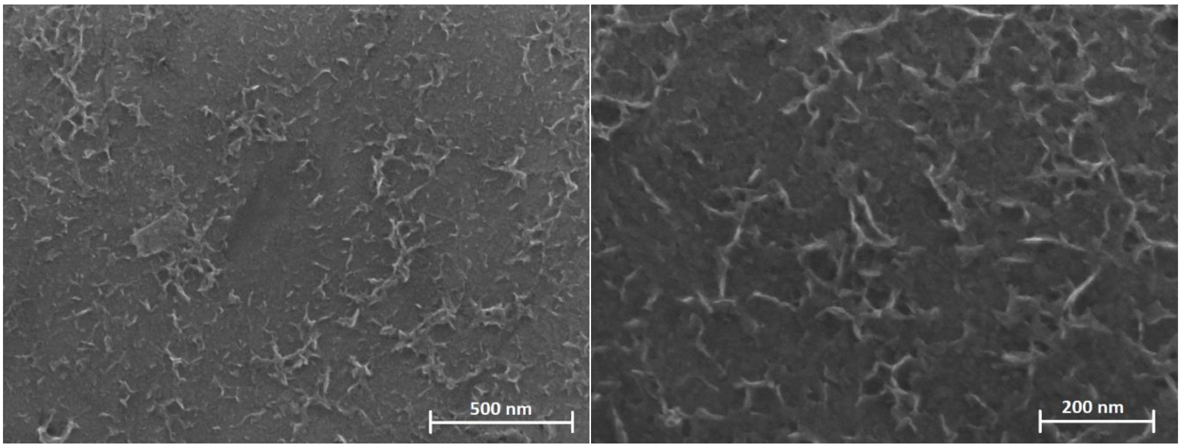

Figure S4. SEM images of $Ni(OH)_2$ after conditioning.

XPS of $Ni(OH)_2$

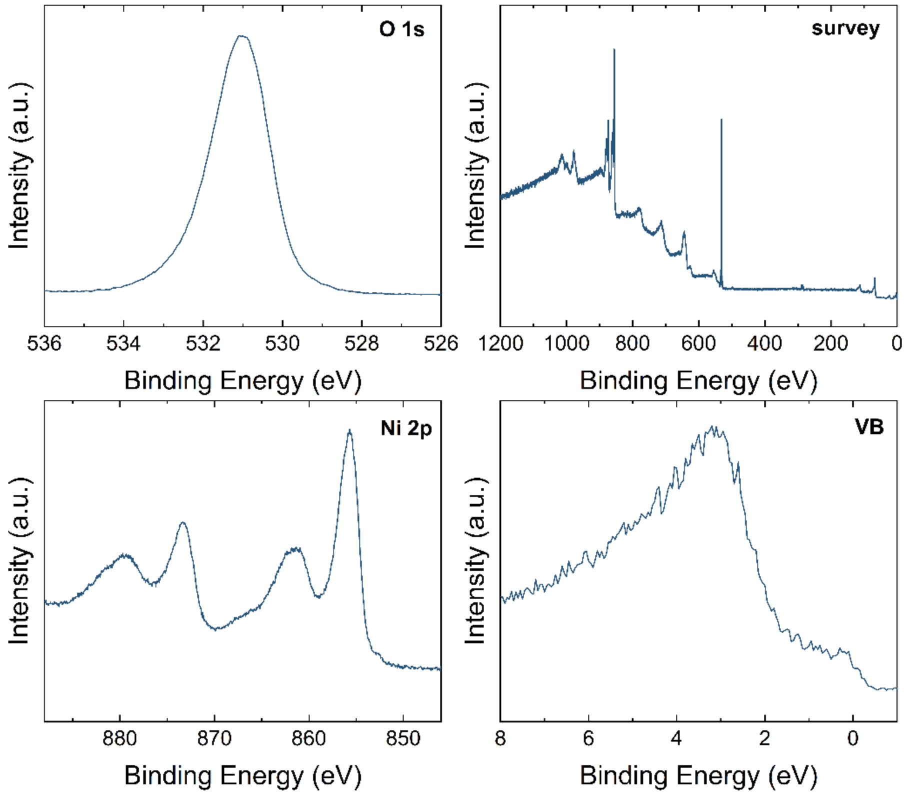

Figure S5. XPS spectra of the $Ni(OH)_2$ phase after conditioning.

The XPS spectra taken after the conditioning show that a clean $Ni(OH)_2$ phase was formed. Both the position and shape of the O 1s and Ni 2p core level peaks confirm the $Ni(OH)_2$ phase. The survey spectrum shows no contamination. The small feature at 852.7 eV in the Ni 2p and the VB intensity at 0 eV show a small spectral contribution of the Ni metal substrate.

XPS after a precisely controlled transfer time

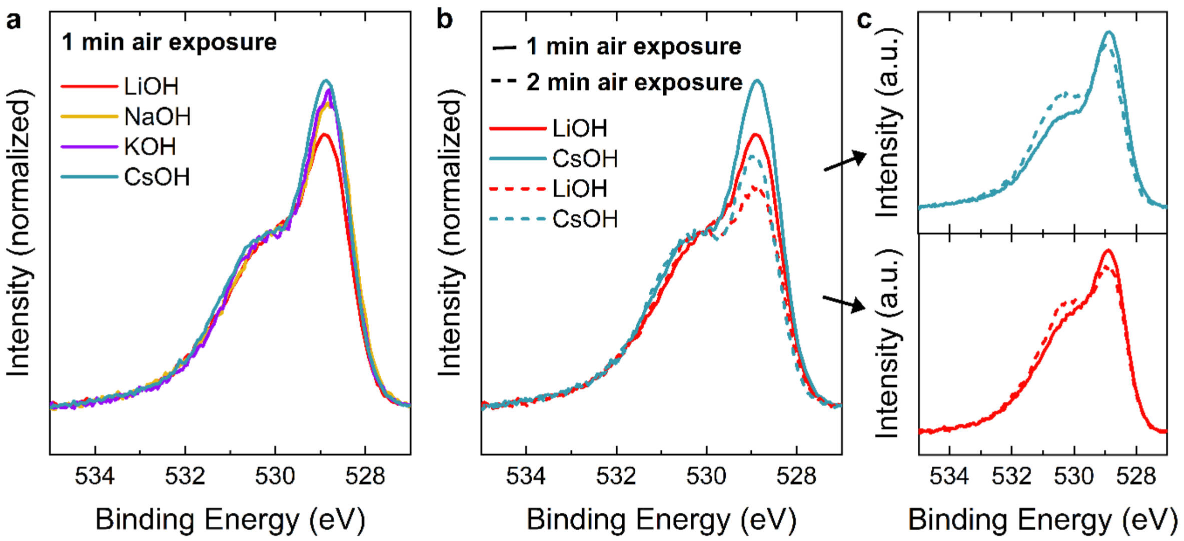

Figure S6. a) O 1s spectra after activation in LiOH, NaOH, KOH, and CsOH and a well-defined transfer time between electrochemical activation and stabilization of NiOOH in UHV. b) Normalized, and c) not-normalized O 1s spectra after activation in LiOH and CsOH and 1 min vs 2 min air exposure.

For the spectra in Figure S6, 4 samples were treated according to the protocol in Figures S2a and S2b. For all samples, the activation in LiOH, NaOH, KOH, and CsOH was done immediately after the conditioning in KOH has finished. After the activation of NiOOH, the potential was stopped and the samples were taken out of the electrochemical cell. From the point of stopping the polarization at 1.6 V, the time was stopped to ensure that all samples were exposed to reducing conditions at OCP for the same amount of time. After stopping the polarization, the samples were immersed for 1 min at OCP in the electrolyte, while the electrochemical cell was disassembled. Next, the samples were rinsed for 10 s. Adjusting the hold on the sample and blow-drying it with nitrogen took 20 s. Finally, the dry sample was exposed to air for 1 min until it was stabilized in UHV. In Figure S6a, a cation dependence of the peak at 528.9 eV is evident. All lines are an average of 2-4 measurements at different positions on the sample surface. The spectra are normalized at 529.8 eV.

To evaluate the impact of different transfer times for the specific samples, two samples, activated in LiOH and CsOH respectively, were measured with XPS after 1 min in air, then exposed to a second minute in air, and measured again with XPS. Figure S6b, shows the resultant decay of the peak at 528.9 eV. In Figure S6c, these spectra are shown without normalization. Clearly, the decay of the peak at 528.9 eV is caused by an increase in OH groups at 530.3 eV. Similarly to the results from water adsorption inside UHV, analyzed in Figure 5, a stronger relative increase in hydroxylation is observed for the sample activated in CsOH.

To conclude, the cation effect on the deprotonation of NiOOH, which is reflected in the peak at 528.9 eV in the O 1s, was confirmed in a control-experiment with fixed transfer times for all samples. The spectra in Figure 2 and Figure 5 in the article might be impacted by small variations in transfer times. In any case, the correlation of a stronger deprotonated NiOOH surface with a stronger OH adsorption holds. With the control experiment also the cation effect on this observation is supported. Nevertheless, it would be a valuable experiment in the future, also for other research questions, to design a (quasi) in situ experiment where the O 1s peak could be measured without transfer through air after the electrochemical activation.

Component fitting of the O 1s core levels

Figure S7 shows an exemplary fit how the O 1s core levels were fitted to obtain the change in Ni-O and Ni-OH groups shown in the article in Figure 5c. The contribution of oxygen-bound carbon groups was omitted in the fitting, as the carbon content was similar across all samples and only the relative changes of the components were of interest, but not absolute numbers for the composition. Table S2 shows the full width half maximum (FWHM) and the binding energies (B.E.) used for the fit. The FWHM of the Ni-OH component is rather large, as the component includes all possible variants of OH bonds, e.g. in NiOOH, $Ni(OH)_2$ or adsorbed on the surface. While the position of the Ni-O and H_2O component was kept constant, the position of the Ni-OH is shifting to higher binding energies with increasing water adsorption.

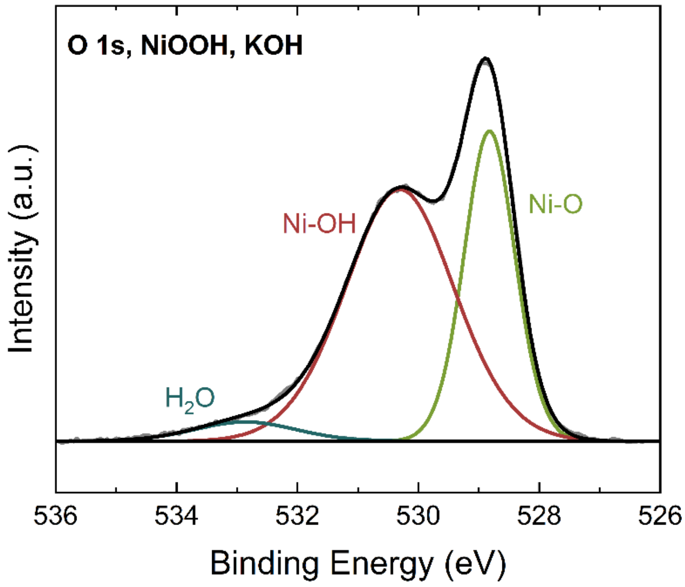

Figure S7. Fitted O 1s core level spectrum of NiOOH activated in 1 M KOH.

Table S2. Fitting parameters for the O 1s core levels.
FWHM = Full Width Half Maximum in eV, B.E. = Binding Energy in eV.

			LiOH	NaOH	KOH	CsOH
		FWHM	B.E.	B.E.	B.E.	B.E.
**Ni-O**		0.96	528.9	528.9	528.8	528.8
**Ni-OH**	NiOOH	2.18	530.3	530.4	530.3	530.4
	15 s 10^{-3} mbar		530.3	530.4	530.3	530.4
	15 min 10^{-3} mbar		530.3	530.5	530.4	530.4
**H_2O**		2.00	532.9	532.9	532.9	532.9

UPS valence band spectra

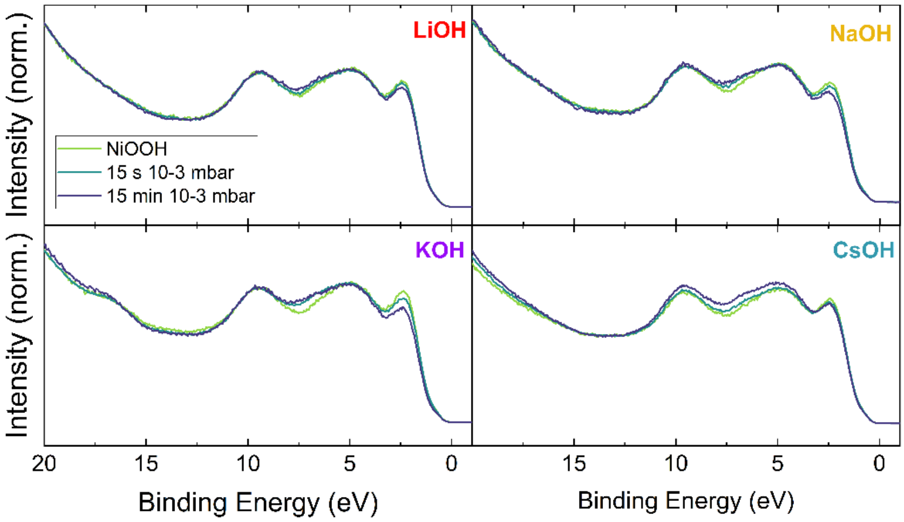

Figure S8. He II valence band spectra of NiOOH activated in 1 M LiOH, NaOH, KOH, and CsOH. The evolution with H_2O adsorption is shown.

pH of used electrolyte solutions

Table S3. pH of the used electrolyte solutions.

	**pH**
LiOH	11.9
NaOH	13.2
KOH	13.7
CsOH	13.8

The pH of LiOH is probably underestimated due to the small size of Li^{+} that negatively affects the measurement with the pH meter.[61]
